# Comparative analysis of naive, primed and ground state pluripotency in mouse embryonic stem cells originating from the same genetic background

**DOI:** 10.1038/s41598-018-24051-5

**Published:** 2018-04-12

**Authors:** Sabitri Ghimire, Margot Van der Jeught, Jitesh Neupane, Matthias S. Roost, Jasper Anckaert, Mina Popovic, Filip Van Nieuwerburgh, Pieter Mestdagh, Jo Vandesompele, Dieter Deforce, Björn Menten, Susana Chuva de Sousa Lopes, Petra De Sutter, Björn Heindryckx

**Affiliations:** 1 Ghent-Fertility and Stem Cell Team (G-FaST), Department for Reproductive Medicine, Ghent University Hospital, Corneel Heymanslaan 10, 9000 Ghent, Belgium; 2 Department of Anatomy and Embryology, Leiden University Medical Center, Einthovenweg 20, 2333 ZC Leiden, The Netherlands; 3 Department of Pediatrics and Medical Genetics, Ghent University Hospital, Corneel Heymanslaan 10, 9000 Gent, Belgium; 4 Laboratory of Pharmaceutical Biotechnology, Faculty of Pharmaceutical Sciences, Ghent University, Ottergemsesteenweg 460, 9000 Ghent, Belgium

## Abstract

Mouse embryonic stem cells (mESCs) exist in a naive, primed and ground state of pluripotency. While comparative analyses of these pluripotency states have been reported, the mESCs utilized originated from various genetic backgrounds and were derived in different laboratories. mESC derivation in conventional LIF + serum culture conditions is strain dependent, with different genetic backgrounds potentially affecting subsequent stem cell characteristics. In the present study, we performed a comprehensive characterization of naive, primed and ground state mESCs originating from the same genetic background within our laboratory, by comparing their transcriptional profiles. We showed unique transcriptional profiles for naive, primed and ground state mESCs. While naive and ground state mESCs have more similar but not identical profiles, primed state mESCs show a very distinct profile. We further demonstrate that the differentiation propensity of mESCs to specific germ layers is highly dependent on their respective state of pluripotency.

## Introduction

Mouse embryonic stem cells (mESCs) are derived from the inner cell mass (ICM) of pre-implantation stage embryos^1,2^. mESCs derived in leukemia inhibitory factor (LIF) and serum are representatives of the naive state of pluripotency. The combination of LIF + serum (LS) is used as a “conventional” culture condition for mESCs derivation. mESCs derivation efficiency in LS-conditions is highly strain dependent, with successful derivation only feasible in certain mouse strains^3^. Depending on whether or not mESCs are readily derived in LS-conditions from a strain of mouse, it is either considered permissive or non-permissive. The mouse strain 129 has been the most commonly used permissive mouse model for ESC derivation, as the derivation efficiency from other permissive strains is very low^4^.

Until 2008, the derivation of germ-line competent mESCs from non-permissive mice was not possible. However, using the combination of two small molecule inhibitors PD0325901 (PD) and Chir99021 (CH), referred to as “2i”, ESCs could also be derived from non-permissive mouse strains at a higher efficiency^5,6^. PD and CH are inhibitors of the fibroblast growth factor (FGF) and the glycogen synthase kinase 3 beta (GSK3β) signaling pathways respectively. In mice, pluripotent stem cells can also be derived from the epiblast of post-implantation stage embryos, commonly referred to as epiblast stem cells (EpiSCs). These pluripotent stem cells display primed characteristics and are dependent upon activation of FGF and Activin signaling for their self-renewal^7,8^. Thus, three distinct pluripotent states, namely naive, primed and ground pluripotency states have been defined in mouse.

We have previously shown that inhibition of the transforming growth factor beta (TGFβ) pathway with the small molecule SB431542 (SB) during culture of pre-implantation embryos increases epiblast proliferation^9,10^. Moreover, SB was shown to promote ground state pluripotency in mESCs, with cultures preserving a higher genomic integrity during long term maintenance^11^. Therefore, SB promotes pluripotency in both ESCs and embryos. Although all these states of mESCs (naive in LS, ground in 2i and 2iSB ESCs derived in presence of TGFβ pathway inhibitor), originate from the epiblast of the pre-implantation embryo, they exhibit distinct pluripotency characteristics. Therefore, defining transcriptional properties of these cells in detail would be of tremendous value.

In the past, several studies have compared different states of pluripotency. LS and 2i mESCs were shown to exhibit differential expression of genes regulating both metabolic and developmental processes^12^. However, this study compared 2i and LS mESCs derived from both non-permissive and permissive mouse strains^12^. In the current study, we derived mESCs in the naive, primed and ground state of pluripotency and in 2iSB conditions from the “129” strain of mouse. We characterized their transcriptional landscape by microarray analysis. Given that mESC derivation efficiency is highly strain dependent, we compared the transcription profiles of 2i, LS, 2iSB mESCs and EpiSCs derived from the same permissive strain of mouse. With 2iSB mESCs, we verified the effects of TGFβ pathway inhibition on mESCs pluripotency. As the functional similarities of these cells remain to be elucidated, we strived to examine the differentiation potential of the derived mESCs and assess whether a preferential differentiation-bias towards a specific lineage is observed. Therefore, we compared their functional capacity by differentiating them as embryoid bodies (EBs). We demonstrate that naive, primed and ground state mESCs have distinct transcriptional profiles, while their states of pluripotency directly affect spontaneous differentiation towards certain germ layers.

## Results

### Naive, primed and ground state mESCs exhibit distinct transcriptional profiles

**Figure 1:**
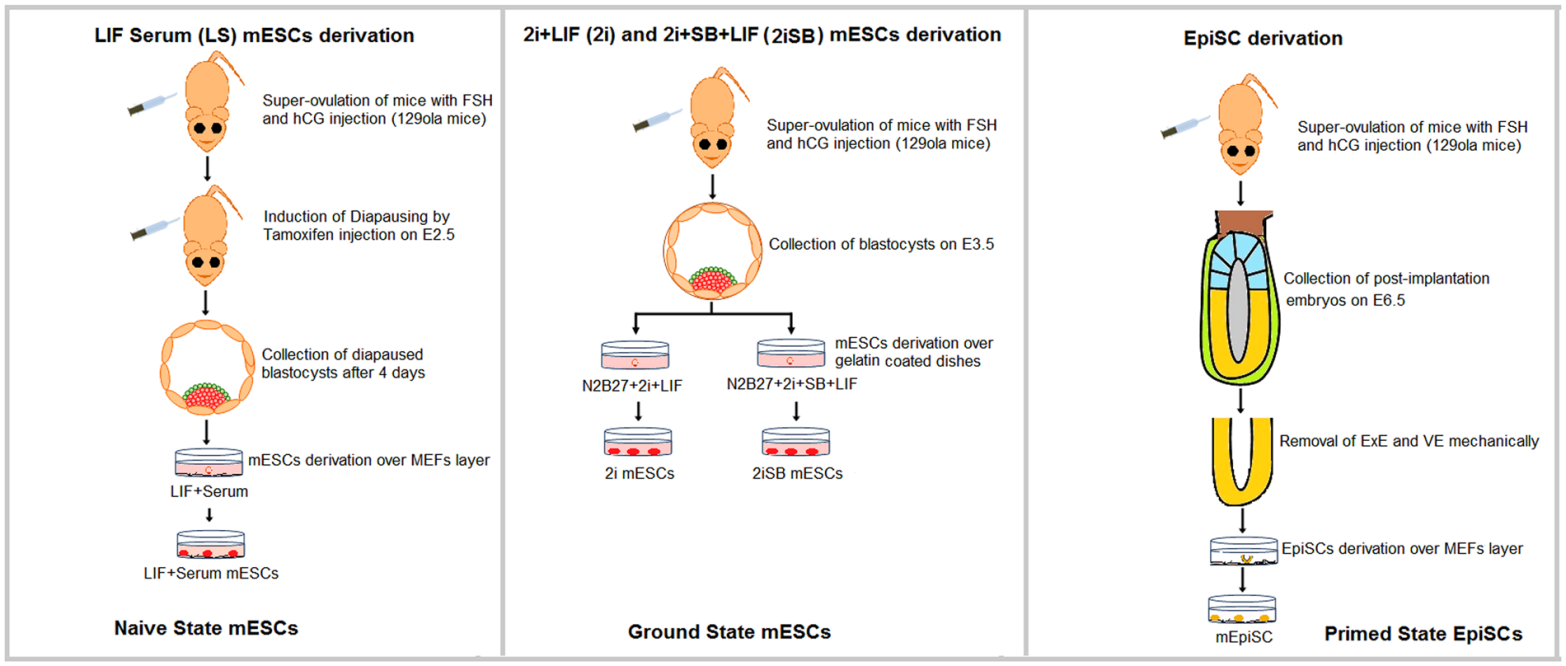
Schematic representation of mouse embryonic stem cell (mESC) derivation in the naive (LS), ground state (2i and 2iSB) and primed state (EpiSCs) of pluripotency. All ESCs were derived from the 129olahsd strain of mice. mESCs in LS, 2i and 2iSB were derived from pre-implantation stage embryos on embryonic day (E3.5) post coitum and EpiSCs were derived from post-implantation stage embryos on E6.5.

**Figure 2:**
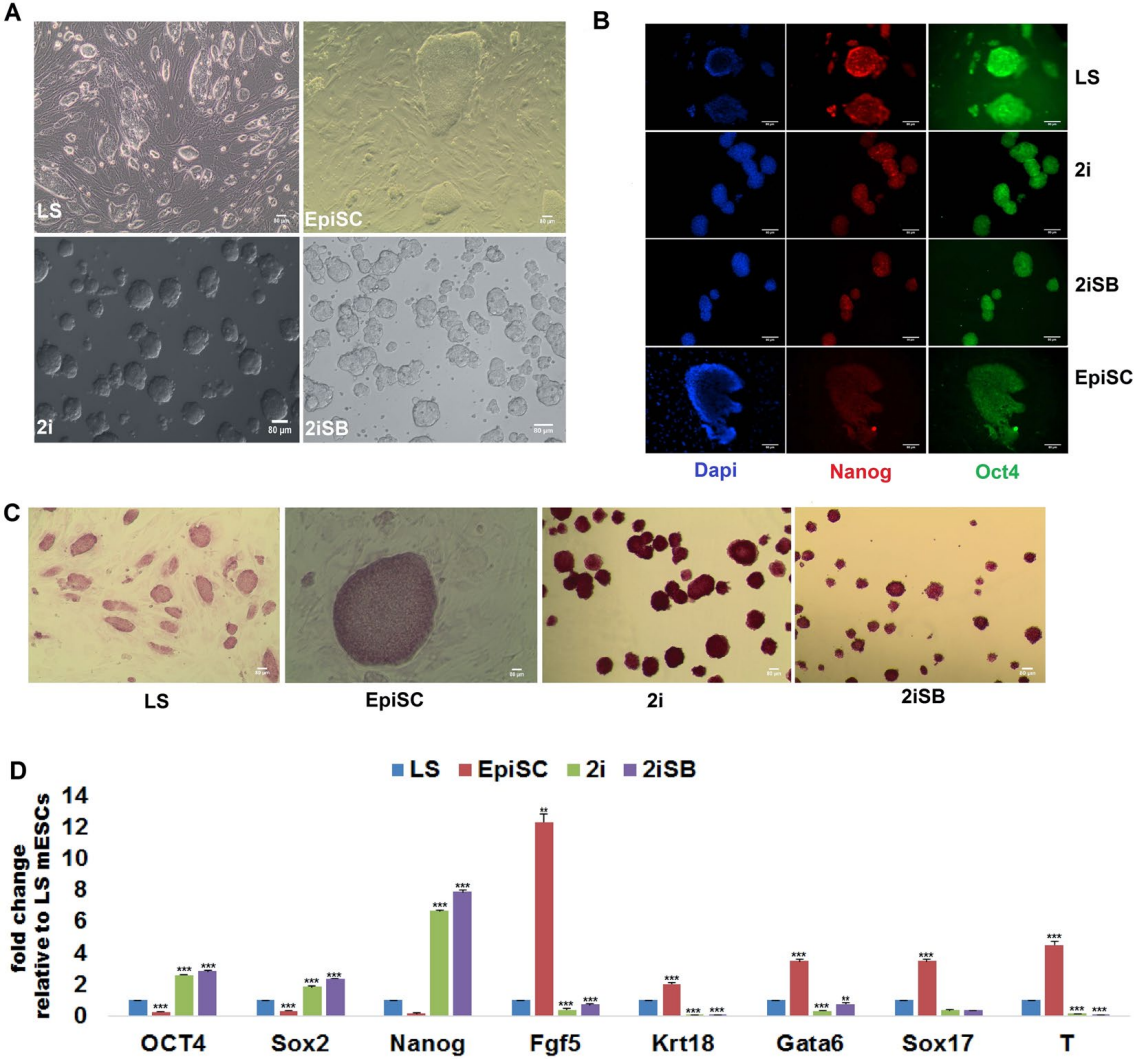
Characterization of LS, 2i, 2iSB mESCs and EpiSC. (**A**) Morphological appearances of naive state (domed colonies); ground state (ball like morphology, as they grow floating) mESCs and primed state EpiSCs (flattened colonies) (**B**) OCT4 and NANOG expression at the protein level (immunofluorescence images) (**C**) AP activity in ESCs in all conditions and (**D**) qPCR for pluripotency and lineage specific markers, Ct values were primarily normalized to the value of *Gapdh* and the expression of all genes in all other samples was compared to LS mESCs gene expression. qPCR was performed in four cell lines from EpiSCs, 2i and 2iSB mESCs and LS mESCs. The average Ct values over the replicates were used to perform the statistical analysis. p-values < 0.05 are represented by *, p-values < 0.01 by ** and p-values < 0.001 by ***. Scale bars represent 80 μm.

mESCs were derived in four different culture conditions: naive state in LIF + Serum (LS) condition and ground state in 2i + LIF (2i) condition from the pre-implantation embryos and primed EpiSCs in Activin A, bFGF and IWP2 condition from the post-implantation stage epiblasts. We also derived mESCs in 2i + SB + LIF (2iSB) culture conditions from preimplantation mouse embryos (Fig. 1). Basic characterization was performed in ESCs derived in all four conditions (LS, 2i, 2iSB and EpiSCs) (Fig. 2). All four EpiSCs lines were chromosomally normal as assessed by sequencing (Table 1). However, in LS, 2i and 2iSB culture conditions, some ESC lines were chromosomally abnormal (Table 1). Trisomy 8, which is frequently observed in mESCs^13^, was present in 5/15 mESC lines (two lines from each of LS, and 2iSB culture conditions and one line from 2i). Trisomy 16 was found in 3/4 lines in 2iSB culture conditions, whereas trisomy 11 in 2/3 LS lines. This indicates that culture conditions might initiate particular chromosomal aberrations in mESCs.

**Table 1:** Summary of the passage number and chromosomal status of cell lines used for microarray analysis and differentiation experiments.

Cell Line	Passage number used for			Chromosomal Status
	Microarray analysis	Differentiation initiation	Sequencing/karyotyping	
2i1	24	19	31	Normal Karyotype
2i2	22	19	31	Trisomy 16
2i3	23	—	28	Trisomy 8 and Trisomy 16
2i4	22	—	8	Normal Karyotype
2iSB1	22	24	37	Trisomy 8
2iSB2	17	12	25	Trisomy 2 and trisomy 16
2iSB4	19	—	26	Trisomy 8, trisomy 16, trisomy 3 and trisomy 18 in subpopulations
2iSB5	23	—	31	Trisomy 2, trisomy 8 and trisomy 16
EpiSCB	24	26	22	Normal Karyotype
EpiSCI	24	21	22	Normal Karyotype
EpiSC4	28	—	24	Normal Karyotype
EpiSCF	24	—	22	Normal Karyotype
LS1	23	23	31	Normal Karyotype
LS2	24	24	32	Trisomy 8 + Trisomy 11
LSFF	26	—	37	Trisomy 8 + Trisomy 11

**Figure 3:**
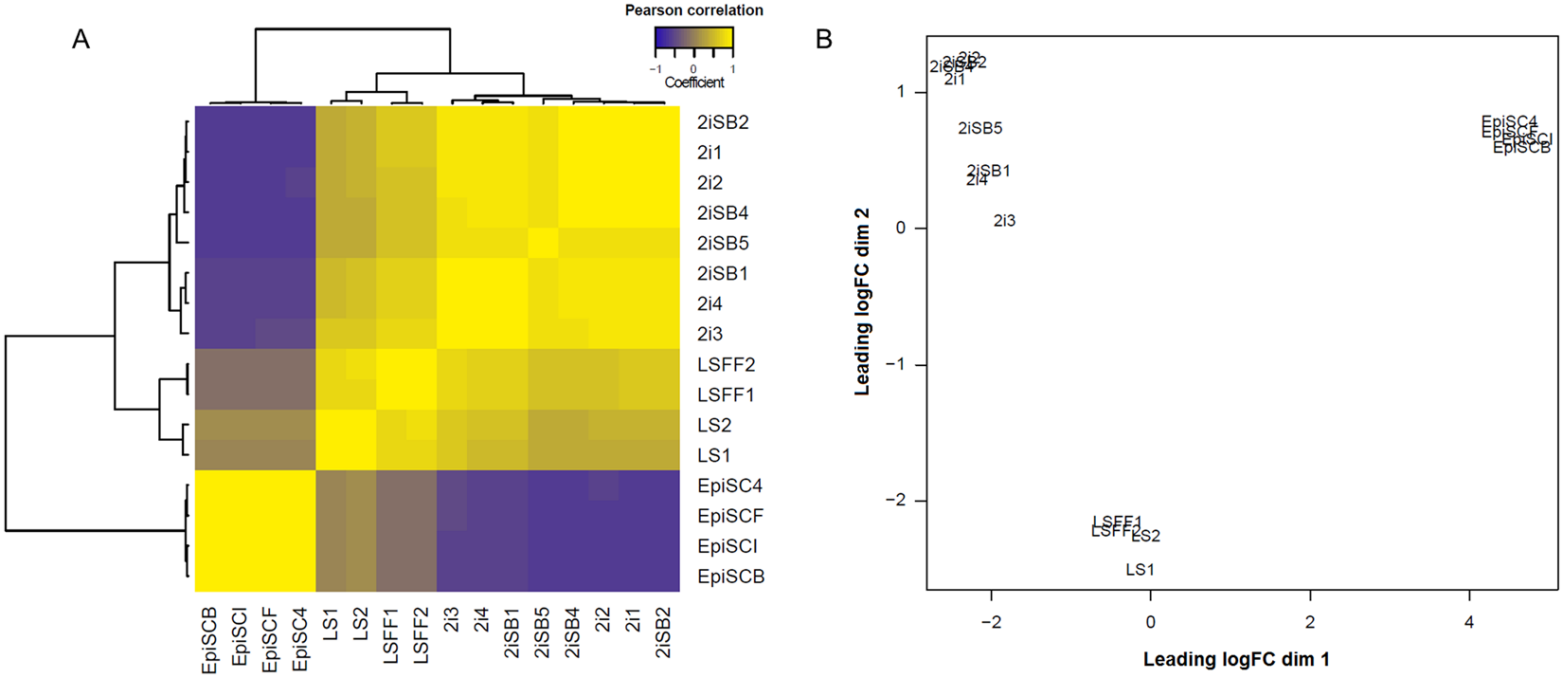
Naive, primed and ground pluripotency represent distinct pluripotency states. (**A**) Heat map showing the Pearson correlation between different states of pluripotency based on the 500 most variable probes with FDR < 0.05, fold change > 2 was considered significant. (**B**) Multidimensional scaling (MDS) plot for mESCs in the naive, primed and ground state of pluripotency. Biological replicates of all samples clustered together in the MDS plot.

Subsequently, we performed Agilent SurePrint G3 Unrestricted Mouse Gene Expression 8 × 60K Microarray analysis for the transcriptional profiling of these cells. Four cell lines from each group, LS (LS1, LS2, LSFF1 and LSFF2), EpiSCs (EpiSC4, EpiSCB, EpiSCI and EpiSCF), 2i (2i1, 2i2, 2i3 and 2i4) and 2iSB (2iSB1, 2iSB2, 2iSB4 and 2iSB5) mESCs were analyzed. The passage number of each line used for microarray analysis has been summarized in Table 1. LS1 and LS2 lines were derived from diapaused embryos, while LSFF1 and LSFF2 are replicates of the same stem cell line derived from a non-diapaused embryo. The Pearson correlation based on the 500 most variably expressed probes (provided as a supplementary data) between all four groups showed that LS, 2i and 2iSB mESCs displayed strong negative correlation with EpiSCs, while naive and ground state mESC showed stronger correlation to each other (Fig. 3A). 2i and 2iSB mESCs displayed the strongest correlation to each other, whereas naive, primed and ground state mESCs exhibited unique transcriptional profiles, distinct from each other (Fig. 3A). A multidimensional scaling (MDS) plot showed 3 distinct clusters for the naive, primed and ground state mESCs, respectively (Fig. 3B). Primed state EpiSCs formed a separate cluster, away from both naive (LS) and ground state (2i) mESCs, illustrating their unique identity – distinct from naive and ground state mESCs. The naive mESCs (LS) and the ground state mESCs (2i) also clustered separately indicating that even they are not identical to each other, despite both being derived from pre-implantation stage embryos (Fig. 3B). 2iSB mESCs also clustered away from EpiSCs and LS mESCs, revealing a unique transcriptional profile, distinct from both primed and naive state mESCs However, they clustered together with 2i, sometimes even overlapping, showing that their transcriptional profiles are very similar (Fig. 3B).

**Figure 4:**
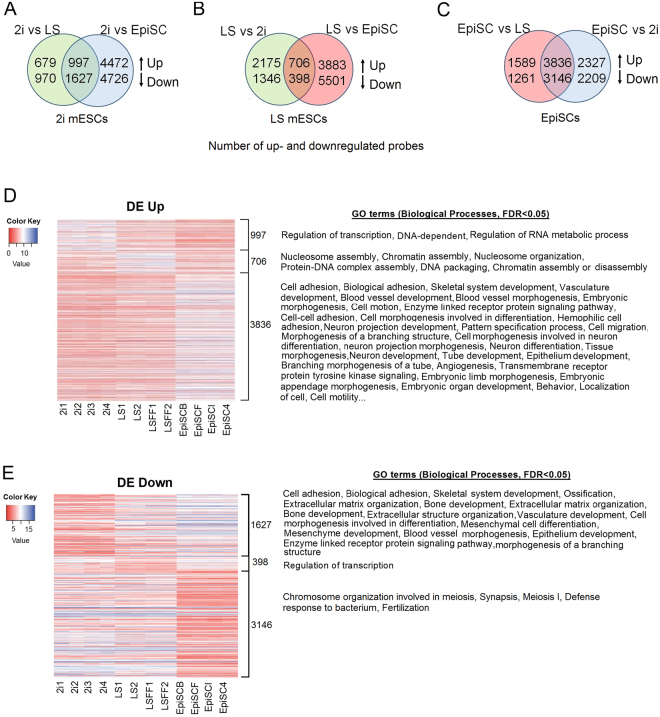
Venn-diagrams showing the number of uniquely up- and downregulated genes per state (FDR < 0.05, fold change > 2). The up arrow denotes the upregulated probes and down arrow denotes the downregulated probes. (**A**) Number of differentially expressed probes for naive (2i) mESCs. (**B**) Number of differentially expressed probes for ground state (LS) mESCs and (**C**) Number of differentially expressed probes for primed EpiSCs. All other duplicate probes were removed keeping the one with highest variance across the sample after analysis. (**D**) Heat map showing the expression levels of the upregulated probes. GO terms that were significantly enriched in the upregulated probes of LS, 2i mESCs and EpiSCs (FDR < 0.05). (**E**) Heat map showing expression levels of the downregulated probes. GO terms that were significantly enriched in the downregulated probes in LS, 2i mESCs and EpiSCs (FDR < 0.05).

We plotted Venn diagrams (Fig. 4A–C) based on differentially expressed probes (FDR < 0.05, fold change > 2) determined by the R package LIMMA to identify the signature of uniquely up- and downregulated genes for each pluripotency state in LS, 2i and EpiSCs. Venn diagrams revealed that EpiSCs have a transcriptional activity that is substantially different from naive and ground state mESCs with 3836 upregulated and 3146 downregulated probes compared to naive and ground state mESCs (Fig. 4C). 2i mESCs had 997 uniquely upregulated and 1627 downregulated probes compared to both EpiSCs and LS mESCs (Fig. 4A) while LS mESCs had 706 upregulated and 398 downregulated probes compared to 2i mESCs and EpiSCs (Fig. 4B). These results confirm the observation from the MDS plot, revealing that the primed state of pluripotency is transcriptionally vastly distinct from naive and ground state mESCs. The smaller number of up- and downregulated genes in the naive versus ground state mESCs revealed that they are more comparable to each other, than to EpiSCs, although they display a unique signature of differentially expressed genes.

### Gene ontology (GO) terms related to metabolic processes, cell-cycle processes and development processes are enriched in 2i mESCs, LS mESCs and EpiSCs respectively

GO analysis showed that compared to the primed EpiSCs and naive LS mESCs, the upregulated genes in the ground state 2i mESCs were predominantly enriched (FDR < 0.05) for GO terms the regulation of transcription and RNA metabolic processes (Fig. 4D), whereas the downregulated genes were enriched in developmental processes (Fig. 4E). In contrast, most of the GO terms that were enriched in significantly downregulated genes in 2i were also enriched in genes significantly upregulated in EpiSCs (Fig. 4D,E). Significantly downregulated genes in primed EpiSCs, compared to 2i and LS mESCs were enriched in GO terms related to meiosis, defense response to bacterium and fertilization (Fig. 4E). Significantly upregulated genes in naive LS mESCs, compared to primed EpiSCs and ground state 2i mESCs, were enriched in GO terms like nucleosome and chromatin assembly (Fig. 4D). Significantly downregulated genes in LS mESCs were enriched in regulation of transcription compared to primed EpiSCs and 2i mESCs (Fig. 4E). List of all differentially expressed probes and enriched GO terms are provided as Supplementary Tables.

### Primed EpiSCs display downregulation of pluripotency genes together with upregulation of lineage specific genes compared to naive and ground state mESCs

**Figure 5:**
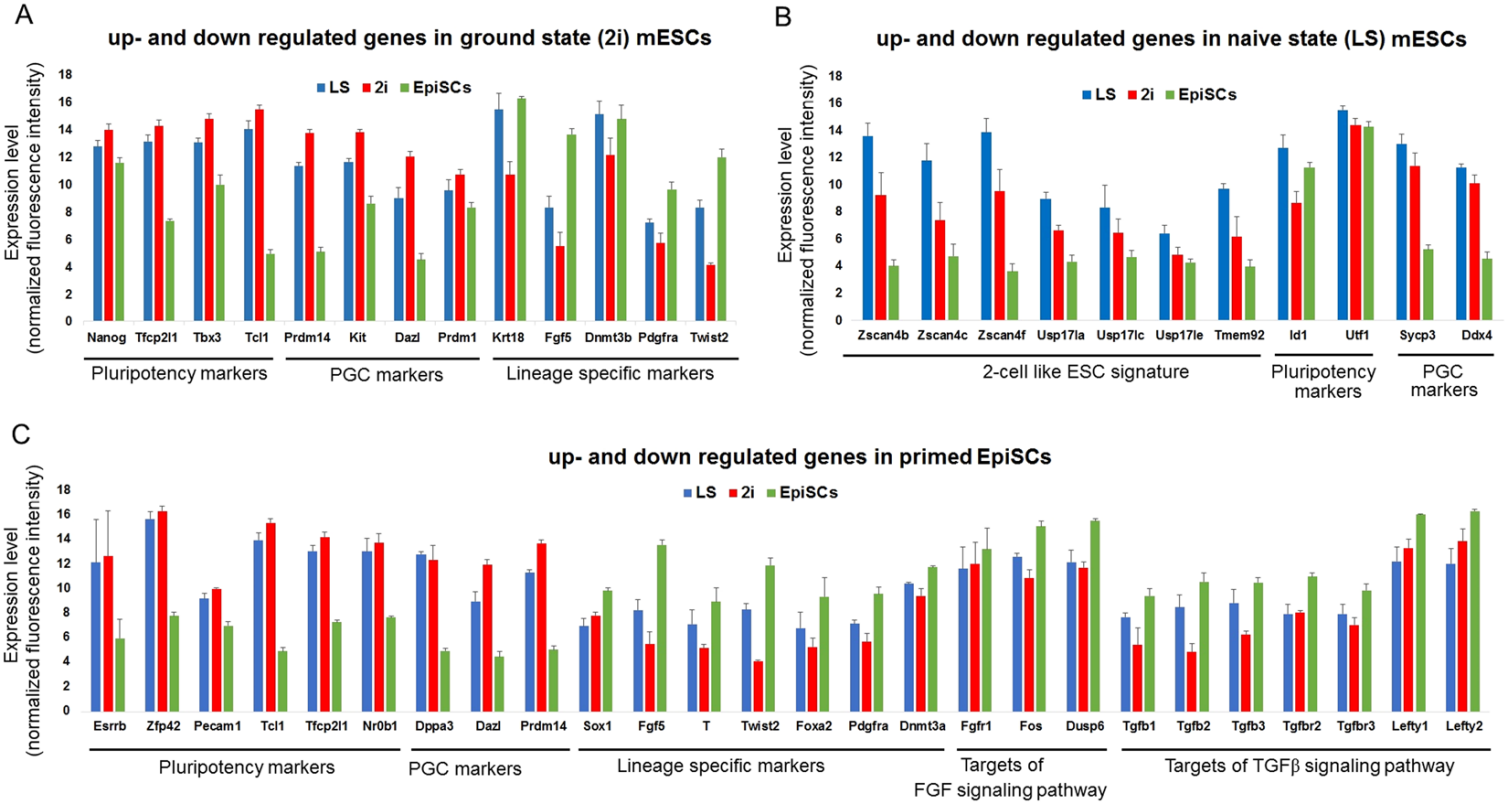
Up- and downregulated genes in naive, primed and ground state mESCs. Expression level (based on the normalized fluorescence intensity) of selected genes from significantly up- and downregulated genes during differentially expressed genes analysis in (**A**) 2i mESCs (**B**) LS mESCs and (**C**) EpiSCs.

Our microarray data revealed that compared to LS mESCs and EpiSCs, 2i mESCs displayed upregulation (FDR < 0.05, fold change > 2) of pluripotency genes, such as *Nanog*, *Tfcp2l1*, *Tbx3*, and *Tcl1* (Fig. 5A) and primordial germ cell (PGC) markers, including *Prdm14*, *Prdm1*, *Kit* and *Dazl*. Lineage specific genes, *Krt18*, *Fgf5*, *Dnmt3b*, *Pdgfra*, *Twist2* etc., were downregulated in 2i mESCs (Fig. 5A) compared to naive and primed ESCs. Genes including the core pluripotency markers – *Nanog* and *Sox2* and the naive pluripotency markers – *Esrrb*, *Rex1 (Zfp42)*, *Pecam1*, *Tcl1*, *Tfcp2l1* and *Nr0b1* and the PGC markers *Stella (Dppa3)*, *Prdm14* and *Dazl* were downregulated in EpiSCs compared to LS and 2i mESCs (Fig. 5A,C). Conversely, lineage specific genes, such as *Sox1*, *Fgf5*, *T*, *Twist2*, *Foxa2*, *Pdgfra* and *Dnmt3a*, targets of FGF signaling – *Fgfr1*, *Myc*, *Fos*, *Dusp6* and targets of TGFβ signaling – *TGFb1*, *TGFb2*, *TGFb3*, *TGFbr2*, *TGFbr3*, *Lefty1*, *Lefty2*, *Inhba*, *Furin* etc., were upregulated in EpiSCs, compared to 2i and LS mESCs (Fig. 5C). A certain population of mESCs in 2i exhibit some 2-cell embryo like transcriptional characteristics^14,15^. Analysis of the 10 most highly expressed genes from these transcripts in a previous study showed that these genes were absent in mESCs grown in LS^14^. In contrast, we observed upregulation of 7 out of 10 of these genes, including *Zscan4b*, *Zscan4c*, *Zscan4f*, *Usp17la*, *Usp17lc*, *Usp17le* and *Tmem92* in LS mESCs, compared to 2i mESCs and EpiSCs (Fig. 5B). Pluripotency maintenance genes, such as *Id1*, *Utf1* and the late PGC markers - *Sycp3*, *Ddx4* were also upregulated in LS mESCs compared to EpiSCs and mESCs in 2i (Fig. 5B).

### mESCs in 2i and 2iSB are comparable

**Figure 6:**
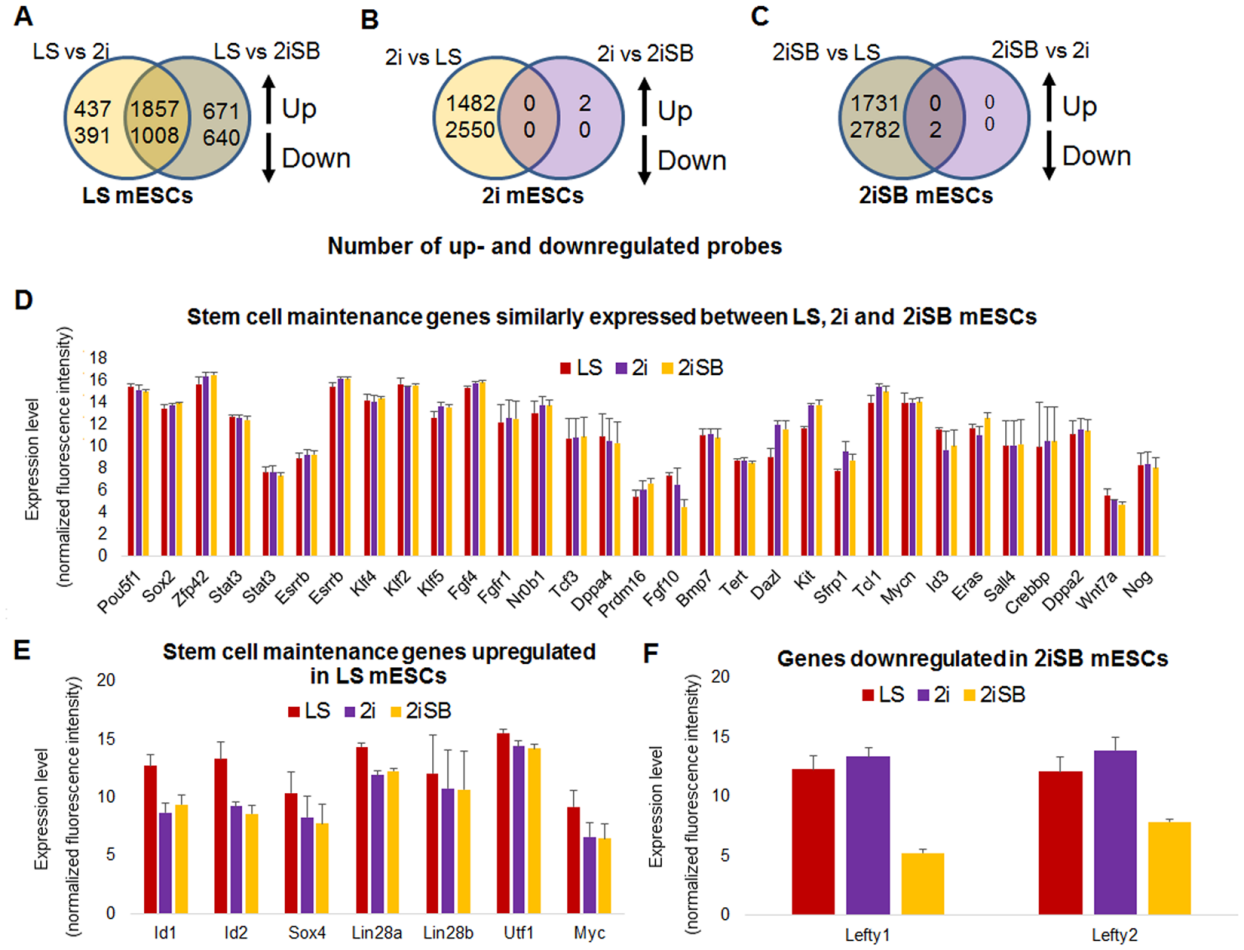
Venn-diagrams showing the number of up- and downregulated probes (values within overlapping sections of each group) in (**A**) LS mESCs, (**B**) 2i mESCs and (**C**) 2iSB mESCs (FDR < 0.05, fold change > 2). The up arrow denotes the upregulated probes and down arrow denotes the downregulated probes. All other duplicate probes were removed keeping the one with highest variance across the sample after analysis. (**D**) Expression level (based on the normalized fluorescence intensity) of similarly expressed genes between LS, 2i and 2iSB mESCs (**E**) significantly upregulated genes during differentially expressed genes analysis in LS mESCs and (**F**) significantly downregulated genes during differentially expressed genes analysis in 2iSB mESCs.

Next, in a separate analysis, we compared differentially expressed genes (FDR < 0.05, fold change > 2) between 2i, 2iSB and LS mESCs. Cell-specific analysis based on differentially expressed genes showed that among these three mESCs, LS mESCs had the highest number of differentially expressed probes, with 1857 upregulated and 1008 downregulated probes, when compared to 2i, 2iSB mESCs (Fig. 6A). This underlines the unique transcriptional identity of LS mESCs, distinct from 2i and 2iSB mESCs. Stemness maintenance genes like *Id1*, *Id2*, *Myc*, *Lin28a*, *Lin28b*, *Utf1*, and *Sox4* were upregulated in LS grown mESCs compared to 2iSB and 2i mESCs (Fig. 6E), while genes like *Oct4*, *Sox2*, *Klf2*, *Klf4*, *Klf5*, *Rex1*, *Esrrb* were similarly expressed in all three mESC culture conditions (Fig. 6D). Conversely, 2i mESCs did not show any unique differentially expressed genes and were almost identical to 2iSB mESCs, which showed two downregulated genes (Fig. 6B,C). *Lefty1* and *Lefty2* were downregulated in 2iSB mESCs, compared to those cultured in 2i and LS (Fig. 6F). Therefore, the mESCs in 2i and 2iSB culture conditions were nearly indistinguishable from each other.

### Differentiation Assay

**Figure 7:**
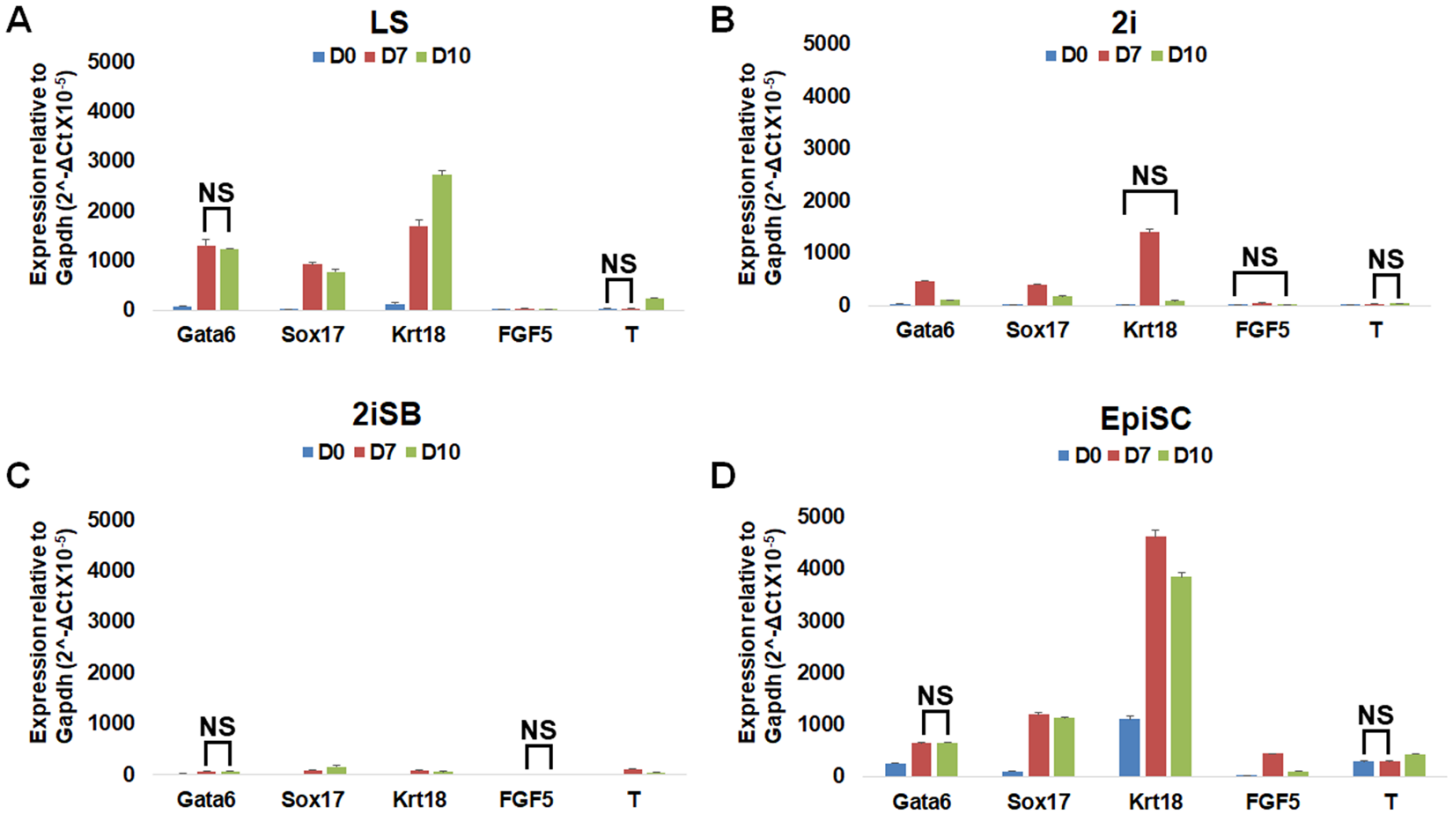
Spontaneous differentiation of LS, 2i, 2iSB mESCs and EpiSCs. Relative expression (in natural log (ln) scale) of ectodermal markers (Krt18 and Fgf5), endodermal markers (Gata6 and Sox17) and mesodermal marker (T) from EBs collected on Day7 and Day10 of spontaneous differentiation of (**A**) LS mESCs (**B**) 2i mESCs, (**C**) 2iSB mESCs and (**D**) EpiSC. CT values were normalized to the value of Gapdh. P-values less than 0.05 were considered significant. Expression of all genes was significantly different on different days (within a particular group) unless otherwise stated as not significant (NS).

mESCs derived in all four conditions (LS, 2i, 2iSB and EpiSCs) were subjected to spontaneous differentiation by embryoid bodies (EBs) formation in their respective basal medium, following the removal of growth factors and/or small molecules. EBs were collected on day (D)7 and D10 of differentiation. Two lines from each four conditions were used for the differentiation experiment. Details of the ESC lines used for differentiation experiments and passage numbers are summarized in Table 1. The expression levels of ectodermal (*Fgf5 and Krt18*), endodermal (*Gata6 and Sox17*) and mesodermal marker (*T*) in the EBs was compared relative to the expression of* Gapdh* in each sample (Fig. 7A–D).

On D0, LS, 2i mESCs, 2iSB mESCs and EpiSCs exhibited different levels of expression of genes associated with different germ layers (Fig. 7A–D). EBs derived from all stem cell conditions, both on D7 and D10 of differentiation, showed significantly elevated levels of most of the lineage specific markers. On D7, in LS and EpiSC EBs, expression was limited to endodermal and ectodermal markers only (Fig. 7A,D). However, expression of the mesodermal marker *T* was not significantly different to its basal level on D0. D7 EBs in 2i exhibited significantly higher expression of all germ layer markers (Fig. 7B). On D10, both LS and EpiSC EBs showed significantly upregulated expression of *T*, which was not significant on D7 of differentiation, together with markers of ectoderm and endoderm lineages (Fig. 7A,D). LS EBs showed downregulation of endodermal markers and upregulation of ectodermal markers on D10 compared to D7 of differentiation, whereas EpiSC EBs showed downregulation of ectodermal markers on D10. 2i EBs on D10 showed downregulation of all germ layer markers except for the mesodermal marker *T* (Fig. 7A,B and D). In 2iSB EBs, although all germ layer markers were significantly upregulated, both on D7 and D10, we observed a reduced differentiation capacity as compared to the ESCs from other culture conditions (Fig. 7C).

## Discussion

Genetic background is essential for stem cell derivation, particularly in LS conditions. Using mouse models with divergent genetic background for studying different states of pluripotency in mESCs is likely to influence research outcomes. To ensure unbiased conclusions, in the current study, we performed microarray analysis to characterize and compare the transcriptional signatures of different states of pluripotency using cells derived from the same mouse strain within the same laboratory. We showed that naive, primed and ground state mESCs have distinct transcriptional profiles. Our findings are in concordance with previous RNA sequencing studies by Marks *et al*.^12^ (RNA sequencing in bulk cells) and Kolodziejczyk *et al*.^14^ (single cell RNA sequencing), who also reported transcriptional variation between ground and naive state of pluripotency. Here, we also report the transcriptional variation in EpiSCs and 2iSB mESCs in addition to 2i and LS mESCs derived from the same mouse model.

Marks *et al*.^12^ compared naive and ground state mESCs from mixed genetic backgrounds and showed that the core pluripotency markers, *Oct4*, *Sox2*, *Klf4* and *Nanog*, among other 15 stem cell maintenance genes were similarly expressed between 2i and LS condition. In contrast, Kolodziejczyk *et al*.^14^ reported a significant upregulation of these 4 genes in 2i grown mESCs. In the current study, we observed that only *Nanog* and *Tbx3* out of the 15 genes, as reported by Marks *et al*.^12^, were upregulated in 2i mESCs compared to LS mESCs and EpiSCs. Furthermore, we observed fewer (7) genes in LS and (3 genes) 2i mESCs involved in stem cell maintenance, with elevated expression. We speculate that discrepancies in results are due to the differences in the genetic background of the mice used for ESCs derivation in the previous study.

Primed EpiSCs are dependent on FGF and Activin signaling for their self-renewal and are characterized by a lower expression of naive markers and a higher expression of lineage specific markers^7,8^. In the current study, we found many targets of both FGF and Activin signaling pathways to be upregulated in EpiSCs lines, highlighting their dependency on them. As expected, we also observed a repressed level of naive markers along with an elevated expression of lineage specific markers in our EpiSCs. Furthermore, we found that primordial germ cell (PGC) markers were downregulated in primed EpiSCs, while most of these were upregulated in ground state mESCs in 2i.

Although, 2i mESCs are considered to be more homogeneous compared to LS mESCs^12^, recent studies have indicated a heterogeneous distribution of transcription factors within subpopulations of these cells^14–16^. Macfarlan *et al*.^15^ reported the existence of a subpopulation of cells in 2i mESCs with 2-cell stage embryos like cells. Kolodziejczyk *et al*.^14^ demonstrated that these 2-cell like cell population only exists in the 2i-conditions and not in LS-conditions. In contrast, we found that 7 out of 10 most significantly expressed transcripts of 2-cell like cells were in fact upregulated in LS mESCs, compared to 2i mESCs and EpiSCs. This discrepancy might have originated due to differences in the source of mESCs or due to the difference in analysis technique (microarray vs sequencing) used between two studies.

Our results demonstrate that mESCs derived in 2iSB culture condition were almost identical to ground state mESCs at the transcriptional level. Only two genes, *Lefty1* and *Lefty2*, were differentially expressed between 2i and 2iSB mESCs. However, to further understand the functional role of these two genes in 2iSB mESCs, it will be interesting to knockdown or knockout these genes and investigate its effect. Inhibition of the TGFβ pathway with SB in mESCs is known to remove the expression of phosphorylated Smad2 (pSmad2) and to repress the expression of both *Lefty1* and *Lefty2*, along with the repression of lineage specific markers in mESCs^17,18^. Moreover, knockdown of Smad2 maintains mESC pluripotency via enhanced expression of Inhibitor of differentiation (*Id*) genes^18^. *Lefty1* and *Lefty2* maintain the balance between self-renewal and differentiation of mESCs^19^. Thus, we may conclude that 2iSB condition in fact represents the ground state of pluripotency, rather than a distinct state.

From our differentiation experiments, we observed that the cells in different states of pluripotency also differ in their propensity to differentiate towards the ectodermal, endodermal and mesodermal lineages. However, instead of forming EBs by spontaneous differentiation, it would be interesting to see if these cells would behave similarly during directed differentiation towards specific lineages. Furthermore, other techniques, such as immunostaining and flow cytometry could be used to see if the expression of the specific germ layer markers at mRNA level corresponds with their expression at protein level. Upon spontaneous differentiation of mESCs in LS and EpiSCs, increased expression of lineage specific markers was observed compared to spontaneous differentiation of 2i and 2iSB conditions. Similarly, GO analysis showed that genes upregulated in LS and EpiSC conditions were enriched for developmental processes. However, the genes upregulated in 2i mESCs were enriched in the processes like regulation of transcription and RNA metabolic processes. 2iSB mESCs were almost identical to 2i mESCs transcriptionally as only two genes were significantly different between these two. This may explain why we observed enhanced expression of lineage specific markers in LS and EpiSCs compared to 2i and 2iSB mESCs. This may further indicate that mESCs in LS and EpiSCs could be already predisposed for lineage-specific differentiation. However, 2i and 2iSB mESCs may require more time to exit from their ground pluripotent state and only initiate proper differentiation when exposed to prolonged differentiation protocols. A recent study by Choi *et al*.^20^ demonstrated that prolonged culture of mESCs in presence of PD impaired the developmental potential of these cells^20^. Therefore, the lower level of different germ layer markers in our 2i and 2iSB-derived EBs could be due to the continuous presence of PD in the culture medium from derivation onwards.

We can conclude that the naive, primed and ground states of pluripotency have unique transcriptional profiles. Furthermore, inhibition of the TGFβ pathway supports naive pluripotency via inhibition of *Lefty1* and *Lefty2*. Finally, pluripotent stem cells show variation in the level of differentiation to ectoderm, endoderm and mesoderm lineages depending on their respective pluripotency state.

## Materials and Methods

All products used in the experiments were purchased from Sigma unless otherwise stated. All animal experiments were approved by and were performed in accordance to the guidelines and regulations of the Animal Ethics Committee of Ghent University Hospital, Belgium (ECD number 12/61). All experiments were carried out in “129P2olahsd (129)” mice (Harlan Laboratories, The Netherlands). Embryos used in the study were recovered from superovulated female mice, for which 7.5IU of follicular stimulating hormone and 7.5IU of human chronic gonadotropin were injected intraperitoneally, 46–48 hours apart.

### Small molecules and growth factors

Small molecules used in this study are inhibitor of fibroblast growth factor (FGF) signaling, PD0325901 (PD, 1 µM, Cayman), inhibitor of glycogen synthase kinase (GSK) signaling, chir99021 (CH, 3 µM, Axon Medchem), inhibitor of transforming growth factor beta (TGFβ) signaling, SB431542 (SB, 10 μM, Tocris) and an inhibitor of Wnt signaling (IWP2, 2 µM). The combination of PD and CH is termed “2i” and the combination of PD, CH and SB is termed 2iSB. The cytokine Leukemia Inhibitory Factor (LIF) was used during derivation and culture of mESCs from pre-implantation embryos, whereas basic fibroblast growth factor (bFGF) and Activin A were used during derivation and culture of EpiSCs.

### mESCs derivation and culture in LIF-serum

E3.5 blastocysts from 129 mice were used for derivation of mESCs in LIF-serum (LS) condition. For embryo collection, female mice were superovulated by intraperitoneal injection of 7.5IU follicular stimulating hormone (FSH), followed by 7.5IU human chorionic gonadotrophin (hCG), 46–48 hours later. Females were kept with males after the second injection to set-up the mating. Blastocysts were collected on E3.5 days post coitum (dpc) by flushing the uterine horns. The embryos were subsequently cultured overnight in Cook Blastocyst medium to allow spontaneous hatching. The following day, hatched blastocysts were plated in gelatin coated centre-well dishes (BD Biosciences) containing LS medium, consisting of DMEM, 20%FBS, L-glutamine, penicillin-streptomycin, NEAA, β-mercaptoethanol (Gibco) and 1000units/ml LIF (Millipore). The ICM outgrowths were trypsinized after 3 days and re-plated in a fresh culture dish. The ESC colonies were picked manually by scratching with pulled pipettes and were subsequently trypsinized. The ESCs were passaged every alternate day.

For induction of diapausing in embryos, tamoxifen (10µg/mouse, Sigma) was injected intraperitoneally on E2.5 together with subcutaneous injection of Depo-provera (3mg/mouse, Sigma) to maintain the pregnancy. Diapaused blastocysts were collected after 4 days and were plated for ESCs derivation as described above. However, instead of gelatin, mitomycin C treated mouse embryonic fibroblasts (MEFs) were used as a feeder layer for derivation of mESCs from diapaused embryos.

### mESCs derivation and culture in 2i + LIF and 2iSB + LIF

For mESCs derivation in 2i (PD and CH) and 2iSB (2i + SB), mice were superovulated as described above. Blastocysts were collected on E3.5 and allowed to hatch as outlined previously. The zona free blastocysts were plated in gelatin coated centre-well dishes in N2B27 medium^22^, supplemented with either 2i or 2iSB and LIF. The N2B27 medium consisted of 50% DMEM-F12, 50% Neurobasal medium, L-glutamine, penicillin-streptomycin, NEAA, β-mercaptoethanol, N2 supplement, B27 supplement (Gibco) and bovine serum albumin (BSA, Calbiochem). The ICM outgrowths were trypsinized after 3 days and re-plated in a fresh culture dish. The ESCs were passaged every alternate day.

### EpiSCs derivation and culture

EpiSCs were derived as previously described^21^ with some modifications. E6.5 embryos were collected from superovulated female mice. Extra embryonic ectoderm and visceral endoderm were removed with the help of 25 G needles. Then the remaining epiblast was plated over the MEF feeder layer in a centre-well culture dish containing EpiSCs medium. The EpiSCs medium consisted of knockout-DMEM, 20% knockout serum replacement, NEAA, penicillin-streptomycin, L-glutamine, β-mercaptoethanol, bFGF (12 ng/ml), Activin A (20 ng/ml) and IWP2 (2 μM). Three days later, the outgrowths were passaged mechanically using 25 G needle and the clumps of disaggregated cells were seeded onto a fresh feeder layer. The cells were passaged every 2 to 3 days. After 4–5 mechanical passages, for the maintenance of EpiSCs, the cells were passaged every alternate days using CTK solution, prepared as described elsewhere^21^.

### Immunostaining

mESCs were fixed in 4% paraformaldehyde (PFA) for 20 minutes at room temperature (RT). Then the mESCs were washed three times for 5 minutes in PBS at RT and permeabilized in PBS + Triton-X for 8 minutes. After washing mESCs in PBS, they were kept in blocking solution (PBS + BSA + tween-20) for one hour at RT. mESCs were then incubated overnight in primary antibodies, Oct4 (1:200, Santa Cruz, SC-5279) and Nanog (1:100, Abcam, ab80892) at 4 °C. The following day, mESCs were washed and incubated in secondary antibodies, donkey anti rabbit alexa fluor 594 (Abcam, 1/500) and donkey anti mouse alexa fluor 488 (Abcam, 1/500) for one hour at RT. mESCs were washed further prior to mounting in Vectashield mounting medium with Dapi (Vector Laboratories) and visualized using a fluorescence microscope (Olympus 1 × 71, Olympus, Aartselaar, Belgium).

### Alkaline Phosphatase (AP) staining

AP staining was performed according to the manufacturer’s instructions (Merck Millipore, SCR004). Briefly, mESCs were washed in PBS and fixed in 4% PFA for 2 minutes at RT. After rinsing the cells in TBST solution, they were exposed to the staining solution for 15 minutes at RT. Subsequently, mESCs were washed in TBST solution and overlaid with PBS for visualization under a microscope (SC-30, Zeiss).

### EB formation

For EB formation, mESCs were trypsinized and plated in low-attachment 24-well plates (Co-star). mESCs in the LS condition were differentiated^22^ in LS medium without LIF, whereas mESCs 2i or 2iSB were differentiated in N2B27 medium without the addition of small molecules and LIF. For differentiation of EpiSCs, bFGF, Activin A and IWP2 were removed from the EpiSCs medium. EBs were refreshed every alternate day and collected on Day7 and Day10 of differentiation.

### Real Time quantitative Polymerase Chain Reaction (RT-qPCR)

Samples were collected and lysed in Trizol (Invitrogen). The RNeasy mini kit (Qiagen) was used for mRNA extraction and iScript^TM^ cDNA synthesis kit (Biorad) for cDNA conversion. cDNA was quantified with Qubit® ssDNA Assay Kit on Qubit® 1.0 Fluorometer (Invitrogen). RT-qPCR was performed in triplicate samples on a LightCycler® 480 System (Roche) using iTaq Universal SYBR GREEN supermix (Biorad). The expression of genes was normalized to *Gapdh*. The relative gene expression was compared by one-way analysis of variance (ANOVA) followed by Tukey post-test using IBM^®^ SPSS^®^ Statistics Software.

### Microarray

Gene expression analysis was performed using Agilent SurePrint G3 Unrestricted Mouse Gene Expression 8 × 60 K Microarrays (G4858A-028005). Labeling and hybridization of the samples was performed according to the manufacturer’s protocol (Agilent). After scanning the hybridized slides using the Agilent G2565CA Microarray Scanner and Scan Control Software 8.5, probe intensities were extracted using the Feature Extraction Software (Agilent). Probe intensities were background corrected and normalized using the LIMMA package in R, and the control and low expression probes were removed^23^. Up- and downregulated probes were also identified with LIMMA. All other duplicate probes were removed from the list of up- and downregulated signature probes in each group keeping one with the highest variance across all samples after the analysis. Gene ontology term enrichment was tested with DAVID using all Agilent probes as background^24^. The raw data are deposited on Gene Expression Omnibus (GEO) and can be assessed through GSE78144.

## Electronic supplementary material

Supplementary Dataset 1
